# Temporal trends and clonal diversity of penicillin non-susceptible pneumococci from meningitis cases from 1996 to 2012, in Salvador, Brazil

**DOI:** 10.1186/s12879-015-1049-y

**Published:** 2015-07-30

**Authors:** Milena Soares dos Santos, Jailton Azevedo, Ana Paula de Oliveria Menezes, Soraia Machado Cordeiro, Eliane Cunegundes Escobar, Josilene Borges Lima, Leila Carvalho Campos, Maria da Glória S. Carvalho, Mitermayer G. Reis, Albert I. Ko, Joice Neves Reis

**Affiliations:** Centro de Pesquisas Gonçalo Moniz, Fundação Oswaldo Cruz/ Ministério da Saúde, Salvador, Bahia 40296-710 Brazil; Departamento de Análises Clínicas e Toxicológicas, Faculdade de Farmácia, Universidade Federal da Bahia, 40170115 Salvador, BA Brazil; Instituto Multidisciplinar em Saúde, Campus Anísio Teixeira, Universidade Federal da Bahia, Vitória da Conquista, Bahia 45029-094 Brazil; Universidade Estadual do Sudoeste da Bahia, Jequié, Bahia 45206-190 Brazil; Instituto de Ciências da Saúde, Universidade Federal da Bahia, Salvador, Bahia 40110-902 Brazil; Department of Epidemiology of Microbial Diseases, Yale School of Public Health, New Haven, CT 06520 USA; Centers for Disease Control and Prevention, Streptococcus Laboratory, Atlanta, GA 30333 USA

**Keywords:** *Streptococcus pneumoniae*, Antibiotic resistance, Genotype, Surveillance

## Abstract

**Background:**

Hospital-based surveillance for pneumococcal meningitis has been conducted since January 1996 in the city of Salvador, Brazil. The purpose of this study was to describe the temporal evolution of Penicillin Non-Susceptible *Streptococcus pneumoniae* (PNSSP) in regards to serotype distributions and clonal diversity recovered from meningitis cases over 17 years.

**Methods:**

Broth microdilution was used to identify pneumococcal isolates that were PNSSP (Minimum Inhibitory Concentration > 0.12 μg/ml). The annual incidence rate of meningitis cases was calculated. Serotyping was defined using multiplex polymerase chain reaction assays and quellung reaction. Genetic diversity of PNSSP isolates was assessed using both pulsed-field gel electrophoresis (PFGE) and Multilocus Sequence Typing (MLST) analyses.

**Results:**

A total of 854cerebrospinal fluid (CSF) culture pneumococcal isolates were tested by broth microdilution method and serotyped. A total of 173 (20.3 %) were penicillin non-susceptible (PNSSP) (Minimum Inhibitory concentration ≥ 0.12 μg/ml). The annual incidence of meningitis cases declined from 1.65/100,000 population (1996) to 0.2/100,000 population in 2012 and the rate due to PNSSP declined 82 % over the 17-years of surveillance. PNSSP isolates were restricted to 13 serotypes, being the most common ones serotypes14 (45.1 %; 78/173), 23 F (19.1 %; 33/173), 6B (14.4 %; 25/173), 19 F (9.2 %; 16/173) and 19A (5.2 %; 9/173). Among the PNSSP isolates, 94 % had serotypes represented in the 10-valent conjugate vaccine (PCV10). The predominant serotype 14 clonal groups were identified as PFGE group A/multilocus sequence type 66 (ST66) [35.3 % (61/173)] and PFGE group GK/ST156 [4.6 % (8/173)], the latter one associated with high level resistance to penicillin and ceftriaxone.

**Conclusions:**

Our results show sustained reductions in pneumococcal meningitis cases in the Metropolitan region of Salvador from 1996 to 2012. This might reflect a beneficial impact of conjugate vaccines. Continued surveillance and further studies need to be conducted to better understanding on PCV10 vaccine impact.

## Background

*Streptococcus pneumoniae* (pneumococcus) often resides asymptomatically as part of human upper respiratory tract microbiota. It is an opportunistic pathogen that causes infections leading to otitis media, pneumonia, sepsis and meningitis [1]. The World Health Organization estimated that this agent caused476,000 deaths among children less than 5 years in 2008; most of these deaths occur in developing countries [2]. Furthermore, antibiotic resistance which has been shown to be associated with a limited spectrum of serotypes, commonly responsible for invasive disease, may have adverse impact on the epidemiology of pneumococcal disease [3]. Resistant pneumococci have been isolated in all continents. In several settings over 40 % of clinical isolates exhibit multidrug-resistance [4].

The emergence of *S.pneumoniae* with reduced susceptibility to penicillin has been observed in Brazil [5] and in many parts of the world. In addition, reduced susceptibility to extended-spectrum cephalosporins such as ceftriaxone has become a serious problem because it limits the available treatment options for pneumococcal invasive diseases [6]. Penicillin-non-susceptible isolates have been identified among 15 % of the pneumococcal meningitis isolates reported in Salvador, Bahia since 1996 [7]. These isolates are restricted to five serotypes (14, 19A, 19 F, 23 F and 6B). Serotype 14was the main penicillin-non-susceptible serotype within this area, as it was in the U.S. prior to PCV7 implementation [8], and has disseminated to widely separate geographic areas [3].

Widespread use of PCV7 dramatically affect the epidemiology of invasive pneumococcal disease (IPD) and carriage reservoir [9, 10]. The Ministry of Health incorporated PCV7in Brazil in 2001 for groups with special clinical conditions considered at high risk of IPD, as immunodeficiency, asplenia and severe cardio-pulmonary diseases, who received vaccination at special immunobiological reference center and at private clinics. In 2010, nationwide PCV10vaccination started for children less than 2 years of age through the national immunization program [11].

Pneumococcal clones have been shown to switch their capsular serotype by exchanging genetic material with other pneumococci; such changes could affect the ability of conjugate vaccines to control disease and could alter the relationship between antimicrobial resistance and serotype [1, 3]. Pneumococcal strain surveillance over time is essential to determine the relative importance of established and emerging antimicrobial-resistant clonal complexes [8]. So far, few publications have addressed the distribution of pneumococcal penicillin non-susceptible clonal complexes in Latin America [7]. Here we describe the temporal incidence, serotype distributions and genotype diversity of penicillin-nonsusceptible *S.pneumoniae* (PNSSP) strains recovered from meningitis cases from 1996 through 2012.

## Methods

### Study population and surveillance system

Active surveillance for pneumococcal meningitis was established at the Hospital Couto Maia, a state infectious disease hospital with 120 beds, which serves as the reference center for meningitis in the Salvador Metropolitan region (3,573,973 inhabitants). The state health department requires that all suspected meningitis cases from inside the metropolitan region are referred to that hospital, and more than 95 % of the meningitis reports from the region are reported from that site [12]. From January 1996 to December 2012, our study team reviewed the daily clinical laboratory records at the hospital to identify all patients for whom cerebrospinal fluid cultures yielded *S.pneumoniae*.

### Clinical and epidemiological data collection

For all identified pneumococcal meningitis patients who were admitted to the surveillance hospital, a standardized data entry form was used to extract demographic and clinical information from their medical records. After their cases were identified, patients were interviewed to obtain information regarding potential risk factors for acquiring penicillin-non-susceptible pneumococci, such as acute illness preceding meningitis and comorbidities.

### Laboratory procedures

#### Identification and susceptibility testing

The pneumococcal isolates identified in this hospital were sent to the Laboratory of Pathology and Molecular Biology at the Gonçalo Moniz Research Center CPqGM/FIOCRUZ for confirmation. The microbiological testing to confirm the *S.pneumoniae* isolates were performed by standard methods, including Gram stain, colony morphology on agar media with 5 % of sheep blood, optochin susceptibility (5 μg Oxoid disks) and bile solubility. The broth microdilution method was performed according to Clinical and Laboratory Standard Institute recommendations to determine the susceptibility of the isolates to penicillin, ofloxacin, cefotaxime, clindamycin, chloramphenicol, erythromycin, tetracycline, rifampicin, TMP-SXZ, and vancomycin (Sigma–Aldrich, Germany). Quality control was performed by testing the *S. pneumoniae* ATCC 49619 isolate. The current CLSI criteria were applied for susceptibility interpretation. According to the CLSI breakpoints for parenteral penicillin (meningitis), all isolates with MIC value ≥0.12 μg/mL were defined as PNSSP [13].

#### Determination of capsular serotypes

From 1996 to 2005, pneumococcal strains were serotyped by the Quellung reaction with capsular type-specific anti-pneumococcal sera at the Centers for Disease Control and Prevention (CDC). After 2006, capsular serogroups/serotypes were deduced using multiplex-PCR as described elsewhere [14, 15]. All isolates identified as serogroup 6 in the multiplex-PCR were subjected to wciN6C–specific PCR, as previously described, for the identification of potential serotype 6C and 6D isolates [16]. Isolates with negative or equivocal multiplex PCR results were subjected to Quellung reaction testing for capsular type definition at the CDC.

#### Molecular typing

Briefly, total DNA was extracted and digested with *Sma*I, and the DNA fragments were resolved by PFGE as described previously [17]. The Gel Compar software package (version 4.0; Applied Maths, Bionumerics) was used to compare the band patterns. PFGE patterns were clustered by the unweighted-pair group method using average linkages (UPGMA), and a dendrogram was generated from a similarity matrix calculated using the Dice similarity coefficient, with an optimization of 1.0 % and a tolerance of 1.5 %. PFGE patterns were defined as isolates with a similarity of 80 % or higher in the dendrogram.

MLST was performed as previously described [18] on representative isolates according to the following criteria: (i) a minimum of two isolates from each PFGE pattern containing 10 or more isolates were selected, (ii) a minimum of one isolate from each PFGE pattern containing two to nine isolates was selected and, (iii) for a randomly selected group of 23 isolates with unrelated PFGE patterns.

The sequence types (STs) were obtained with reference at the MLST database (http://pubmlst.org/spneumoniae/) and the alternatives primers recommended by CDC (http://www.cdc.gov/streplab/alt-MLST-primers.html). New allelic profiles have been submitted to the MLST database for ST assignment.

Clonal Complex (CC) are groups of STs which share a recent common ancestor. goeBURST was used to estimate the relationships among the different STs. goeBURST isa java implementation of the eBURST algorithm rules proposed by Feil et al, 2004 [19] that uses a graphic matrix approach to ensure an optimal solution for the placement of links between STs.Version 1.2.1of the software was used (http://goeburst.phyloviz.net).

#### Data analysis

A clinical and epidemiological database was created and analyzed with Epi-Info Version 3.5.1 (CDC, Atlanta, GA). We calculated annual incidence rates (cases per 100,000 population) by dividing the number of cases among residents of the Metropolitan Region of Salvador (MRS) by the estimated population, using 2000 census bureau (3,021,572 population) to calculate rates for 1996 through 2007; for 2008 through 2012, we used the 2010 census estimate of the population (3,573,973 population) [20]. Fisher’s exact or chi-square tests were used to compare differences between proportions for dichotomous variables, and odds ratio (OR) and 95 % confidence interval (CIs) were calculated as measure of association. The chi-square test for linear trends was applied to compare the fluctuations in incidence rates per year and for comparison of PNSSP proportions. Statistical significance was defined as p < 0.05. Univariate and multivariate logistic regression models were constructed to identify risk factors for invasive diseases caused by PNSSP. Multivariate analysis was calculated using unconditional logistic regression. To be included in this model, a variable was considered when presented a *p* value less than 0.05 in univariate analysis.

### Ethics statement

The study was approved by the Institutional Review Board of the Oswaldo Cruz Foundation (FIOCRUZ), Brazilian Ministry of Health and Hospital Couto Maia. Patients were enrolled in the study according to informed consent procedures. All patients or legal guardian gave written informed consent prior to enrolling in the study.

## Results

### Surveillance for penicillin nonsusceptible pneumococcal meningitis

**Fig. 1:**
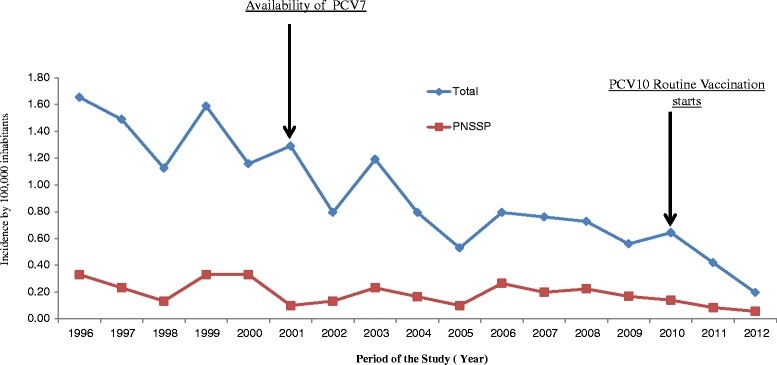
Incidence of pneumococcal meningitis cases in the metropolitan region os Salvador from 1996 to 2012. Abbreviations: Total cases (TOTAL), pneumococcal non-susceptible to penicillin (PNSSP)

A total of 917 patients with *S. pneumoniae* meningitis were consecutively identified at the surveillance hospital during 17 years of laboratory-based active surveillance. Pneumococcal isolates were available for characterization for 854/917 (93.1 %) cases. Considering that 489 patients resided within the Metropolitan Salvador, the annual incidence of pneumococcal meningitis declined from 1.65/100,000 population (1996) to 0.2/100,000 population in 2012. We identified 173/854 (20.3 %) isolates as PNSSP. On the basis of 101(58 %) cases that occurred in patients who resided in MRS and had isolates with penicillin MIC ≥ 0.12 μg/mL, the annual incidence for PNSSP was estimated to be 0.3 case per100,000 population (1996) declined by 82 % in 17-years of surveillance, ranging from 0.3 to 0.06 cases per 100,000 population [*p* < 0.001] (Fig. 1).

### Patient characteristics

Of the 844 patients for whom demographic information was available, 317 (37.6 %) were children aged < 5 years and 542 (64.2 %) were male. Of the 173 cases that were penicillin non-susceptible, 61.1 % occurred in children less than 5 years of age (*p* < 0.05) (Table 1) and 49.1 % occurred in children aged < 2 years. A clinical presentation of meningitis preceded by pneumonia was associated with isolates non-susceptible to penicillin (*p* = 0.03) in univariate analysis (Table 1). The overall case fatality ratio was 30.3 %. After adjusting in multivariate analyses, risk factor for having meningitis due to PNSSP was age less than five years old (OR, 4.2; 95 % CI, 2.1-8.4).

**Table 1:** Characteristics of patients with pneumococcal meningitis, stratified by penicillin susceptibility

Characteristics	Penicillin non-susceptible(*n* = 173)		Penicillin Susceptible(*n* = 681)		OR 95 % CI	*p* value
	N° of cases with information	N° (%) cases	N° of cases with information	N° (%) cases		
Male	170	118 (69.4 %)	671	424 (63.2 %)	1.32 [0.92–1.90]	0.13
Age	162		651			
<5 years		99 (61.1 %)		218 (33.5 %)	4.2 [2.1–8.4]^2^	<0.05
≥5 years		63 (38.9 %)				
Neurological condition at admission^1^	104	81 (77.9 %)	356	289 (81.2 %)	0.82 [0.47–1.40]	0.45
Acute illness preceding meningitis	61		177			
Pneumonia		7 (11.5 %)		7 (3.9 %)	3.15 [1.05–9.37]	0.03
AOM		9 (14.8 %)		31 (17.5 %)	0.81 [0.36–1.82]	0.61
URTI		13 (21.3 %)		38 (21.5 %)	0.99 [0.48–2.01]	0.97
ICU admission	86	38 (44.2 %)	282	137 (48.6 %)	0.84 [0.50–1.36]	0.47
>10 days of hospitalization	157	94 (59.9 %)	643	413 (64.2 %)	0.83 [0.58–1.18]	0.30
Death	160	64 (40 %)	645	195 (30.2 %)	1.54 [1.07–2.20]	0.01

Note. Data are no. (%) of patients for whom information were obtained, *ICU* intensive care unit, *AOM* Acute Otitis Media, *URTI* Upper Respiratory Tract Infection

^1^Coma/Altered mental status. ^2^ Adjusted odds ratio

### Antibiotic susceptibility and serotype distribution

**Fig. 2:**
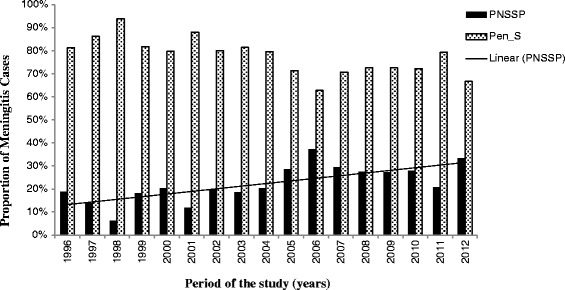
Proportion of penicillin susceptible (Pen_S) and non-susceptible (PNSSP) pneumococcal isolates from 1996 through 2012

All 854 isolates were submitted for antibiotic susceptibility testing and serotyping. All isolates were susceptible to vancomycin, and ≥98 % of isolates were susceptible to ofloxacin, cefotaxime, erythromycin, clindamycin, chloramphenicol, and rifampicin. Overall, 20.3 % (n = 173) of isolates were PNSSP, 48 % (n = 410) to TMP-SXZ, and 27 % (n = 230) to tetracycline. The PNSSP isolates were commonly non-susceptible to TMP-SXZ (138/173, 79.8 %) and tetracycline (27/173, 15.6 %) and less commonly non-susceptible to cefotaxime (7/173, 4.0 %), ofloxacin (3/173, 1.7 %) and erythromycin (1/173, 0.6 %). Resistance to ≥ 3 antibiotics was found in four different serotypes (14, 6B, 23 F and 19 F) (Table 2). Only ten isolates (5.8 %, 10/173) had a high level of resistance to penicillin (three isolates had MIC = 2 μg/mL and seven isolates had MIC ≥ 4 μg/mL). Six of these penicillin resistant strains were also resistant to cefotaxime (MIC ≥ 1 μg/mL). The prevalence of PNSSP isolates increased from 18.8 % in 1996 to 33.3 % in 2012 (chi-square for linear trend = 14.5; p < 0.001) (Fig. 2).

**Table 2:** Characteristics of penicillin non-susceptible pneumococcal isolates from meningitis cases identified from 1996 through 2012

Serotype		Antibiotic resistance	N° of isolates	PFGE profile	ST
VT	14	TMP-SXZ, TET	60	A	66
		CEF	7	GK	156
	6B	TMP-SXZ, CLI, TET	8	AV	751
		ERI, RIF	3	N	4980
	19 F	TMP-SXZ, TET	2	DM	177
		TMP-SXZ	1	GK	156
	23 F	TMP-SXZ, TET, CLI	10	GA	338
		TET, CLO	5	BZ	8080
	9 N	TMP-SXZ	1	GA	737
	7C	TMP-SXZ	1	A	66
	19A	TMP-SXZ	1	DP	1118
			1	N	4926
			1	CI	8098
			1	BQ	2408
NVT	6C	TMP-SXZ	1	FV	2777
	23B	TMP-SXZ	1	AK	3536
			1	FP	8089
	13	TMP-SXZ	1	GD	8094

Note: VT –PCV10 Vaccine Type; NVT –Non PCV10 Vaccine Type; cefotaxime (CEF), clindamycin (CLI), chloramphenicol (CLO), erythromycin (ERI), tetracycline (TET), rifampicin (RIF), trimethoprim-sulfamethoxazole (TMP-SXZ)

**Fig. 3:**
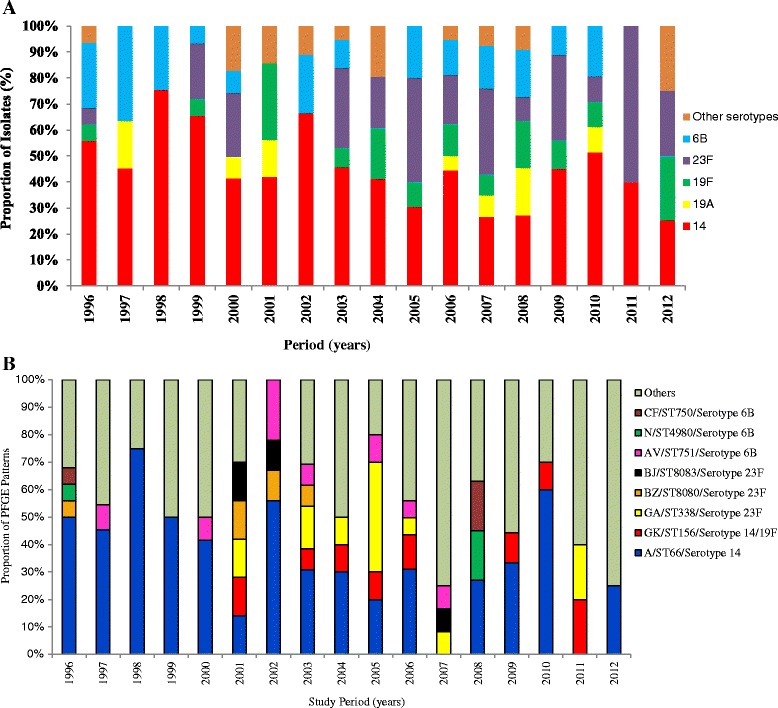
Distribution of most frequent PNSSP serotypes (**a**) and clones (**b**) from meningitis cases identified from1996 through2012. Note (Fig. 3b): Others, includes all PFGE patters with 2 or less isolates

Fifty six capsular serotypes were found among the pneumococcal isolates identified during surveillance. Prevalent serotypes included 14 (12 %), 3 (7.7 %), 6B (7.4 %), 19 F (7.3 %), 23 F (6.6 %), 18C (5.3 %), 4 (4.4 %), 6A (3.4 %), 10A (2.9 %), and 8 (2.4 %). Together, these 10 serotypes represented 512 (60 %) of the isolates. The overall percentage of capsular types included in the PCV10 vaccine accounted for 50.5 %. A total of 13 different serotypes were identified among the PNSSP isolates. The most frequent were 14 (45.1 %; 78/173), 23 F (19.1 %; 33/173), 6B (14.4 %; 25/173), 19 F (9.3 %; 16/173) and 19A (5.2 %; 9/173) (Fig. 3a). With the exception of 19 F, all of these serotypes were significantly associated with penicillin non-susceptibility: 14 (OR, 14.14; 95 % CI, 9.05–22.09), 23 F (OR, 5.49; 95 % CI, 3.20–9.32), 6B (OR, 2.49; 95 % CI, 1.47–4.20) and 19A (OR, 4.07; 95 % CI, 1.60-10.42). Only three serotypes identified among the PNSSP isolates (19A, 12 F and 13) are not represented in the PCV10. The proportions of PCV10 serotypes among the PNSSP isolates were 94 % (163/173). PCV10 projected coverage was 47.9 % (78/163) and 55.2 % (90/163) of PNSSP isolated from patients <2 and < 5 years of age, respectively.

### Molecular typing

A total of 173 PNSSP isolates were analyzed by PFGE and 138were analyzed by MLST. We identified 19 PFGE groups that ranged in isolate number from 2 to 61. A total of 16.8 % (28/173) of the isolates had a single electrophoretic profile. The distribution of the major PFGE clonal groups (composed of 3 or more isolates) is presented in Fig. 3b. Among these, clonal group A was the largest group with 35.3 % (61/173) of the isolates. This clonal group was comprised primarily of serotype 14 isolates and classified as ST66; the second most common group was PFGE type GK /ST 156 with eight isolates, which were mainly serotype 14 (7) and 19 F(1) and resistant to penicillin (MIC range from 2 to 8 μg/mL) and cefotaxime (MIC range 1 to 4 μg/mL). Serotype 23 F also was commonly associated with resistance and was compounded by PFGE type GA/ST 338(10 isolates) followed by PFGE type BZ/ST 8080 (5 isolates) and PFGE type BJ/ST 8083 (3 isolates). Serotype 6Bclustered in three mainly PFGE/MLST types, being PFGE type AV/ST 751 (8 isolates), PFGE type CF/ST4980 (4 isolates) and PFGE type N/ST750 (3 isolates) (Table 1). Additionally, no new genotype was identified within a 2-year period after PCV-10 introduction into the childhood immunization program, at least among PNSSP from meningitis cases.

**Fig. 4:**
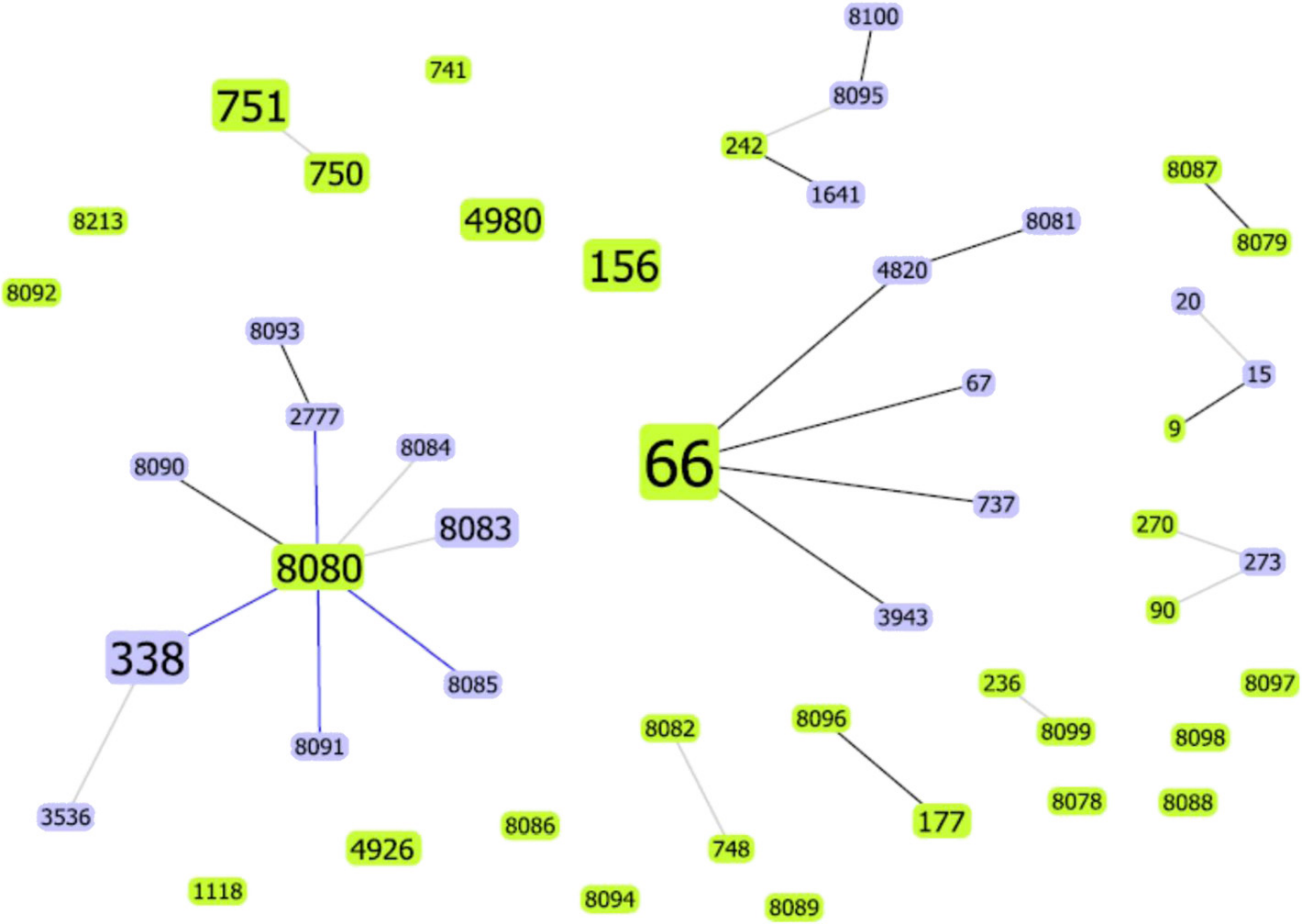
Clonal groups of most frequent PNSSP from meningitis cases. The PMEN- Double Locus Variant clonal groupsofthe138meningitis cases PNSSP isolates and clones (as characterized by MLST). Fifty ST’s were identified and classified into 24 groups. The size of the nodes was proportional to the number of isolates presenting that ST in the database. The ST nodes included: Light green - Group founder; Light blue- Common node; Black - Link drawn without recourse to tiebreak rules; Blue - Link drawn using tiebreak rule 1 and Gray - Link drawn for higher levels (DLV with darker gray or TLV with lighter gray)

The diversity of the 138 PNSSP isolates and the 8 PMEN international clones was visualized with the goeBURST analysis (Fig. 4). As shown in Fig. 4, the STs found in the population of PNSSP isolates were highly diverse and grouped into 24 clusters. The most frequent sequence type among the PNSSP isolates was ST66 serotype 14. This clonal group was also persistent over the 17 years of surveillance.

## Discussion

Since the identification of the first penicillin non-susceptible *S. pneumoniae* case in Brazil in 1988 [21], surveys have reported that up to 20 % of pneumococcal meningitis isolates with decreased penicillin susceptibility [22, 23]. Our data indicate that the proportion of PNSSP in meningitis cases has been fairly stable, with an average of 20.3 % of pneumococcal isolates being non-susceptible to penicillin. In contrast, the pneumococcal meningitis epidemiology continuously changed during the past 17 years in Salvador Metropolitan area. One of the most important changes was the sustained decrease in the incidence rate observed from 1996 to 2012.

In Brazilian nationwide surveillance from 1993 to 2004, an increase of penicillin non-susceptibility was observed (10 % to 28 %), with 6 % of the isolates displaying a high level of resistance [24]. According to Brandileone MCC et al (2006), there are geographic differences between PNSSP isolates in this country. Between 2000 and 2005, in the northeastern region, the proportion of PNSSP isolates was 20 %. The southern and southeastern regions had a higher percentage of PNSSP isolates (28 %) and the lowest rate in the north region was 8 %[24].

The penicillin resistance rate in Brazil was lower than other Latin America countries, including Colombia, Bolivia, Mexico and Venezuela, which reported rates above 30 % [25]. In contrast, Japan have reported even higher proportion of PNSSP (76.6 %) [26].

Additionally, we observed that a high proportion of isolates were non-susceptible to antibiotics commonly used in outpatient settings for acute respiratory infection treatments in Brazil. For example, the proportion of TMP-SXZ non-susceptible isolates reached 72 % in 2006 and keep a rate around 50 % until 2012. In Asian countries invasive pneumococcal isolates remained highly resistant to macrolides, tetracycline, and TMP-SXZ each year [27]. In this study setting, we found a lower proportion (0.6 %) of isolates resistant to erythromycin in comparison with developed countries [28, 29] other Latin American countries [30] and wealthier regions within Brazil [31]. This may be due to the relatively high cost of macrolides, which limits its use in clinical practice in low income population settings.

Serotype distribution information is essential in evaluating the potential benefits of pneumococcal conjugate vaccines, particularly in developing countries where the cost of newly available polysaccharide capsule-protein conjugate vaccines is high relative to the available healthcare resources. Our findings regarding serotype distributions confirm those from national surveys that indicated that a limited spectrum of serotypes were responsible for the majority of meningitis cases due to PNSSP isolates in Brazil. In general, serotypes 14, 23 F, 6B, 19 F and 19A were the most frequent among the PNSSP isolates observed in our study. These results are similar to those reported in other regions in Brazil [32, 33] and in other Latin American countries [6]. Serotype 14 was the most common serotype, which accounted for 44.8 % of the total PNSSP isolates. Additionally, serotypes 23 F, 6B and 19 F are important reservoirs of PNSSP in this setting.

In 2010, PCV10 was introduced country wide for children less than 2 years of age in as part of Brazil’s national immunization program. Recently, the effectiveness of PCV10 in Brazil was evaluating by a case-control study, which demonstrated that PCV10 prevents invasive disease caused by vaccine serotypes, in agreement with our finds in this study [34]. However, evaluation of the serotype distribution showed that serotypes 14, 6B, 23 F, 18C and 19 F remain among the most frequent serotypes causing invasive disease two years after vaccine introduction. As only two years post-vaccination was evaluated (and considering the lower vaccine uptake observed in some places), there may not have been sufficient time to observe the protection effect in all population.

The largest represented clonal group (PFGE pattern A) comprised 35 % of the penicillin non-susceptible isolates. This group mainly included serotype 14 (ST66), which is a single locus variant of the ST67, Tennessee^14^-18clone. ST66 is predominant throughout Brazil and is an important factor for maintaining the penicillin resistance rate [33]. Another important clonal group of serotype 14 is ST156, which emerged in 2001 and had an elevated MIC to penicillin and cefotaxime, this clonal group is a serotype switch variant of clone Spain ^9V^ - 3. ST156 has been associated with a broad variety of serotypes including 6B, 9A, 11A, 14, 15B/15C, 19A, 19 F, 23 F, and 24 F, suggesting a high propensity for recombination events [35]. This clonal complex associated with serotype 14 has been encountered in multiple countries including Norway, France, Spain, and the United States [1, 3, 8]. It is also very important to point out the circulation of Colombia^23F^-26 clone, ST338. This clonal group has also been reported in several countries [30, 36, 37]. Apart from these three clonal groups, the specific clonal structure of the PNSSP isolates was dominated by a few other clones (Fig. 4), highlighting 24 new STs. Such a high genetic diversity seems to be a characteristic of PNSSP clonal complexes in general [1].

Serotype and genotype prevalence fluctuations can occur naturally in pneumococcal populations in the absence of pressure exerted by conjugate vaccines. In the United States, clonal expansion (the increase in the number of previously rare clones that express non-vaccine serotypes) has been documented since the introduction of the heptavalent conjugate vaccine (PCV-7). Non-vaccine serotypes were increasingly a cause of disease in the United States in the post-PCV7 era, most frequently serotype 19A, which is associated with antimicrobial resistance [37]. In Salvador, similar to the findings of others, a relatively small number of serotypes accounted for the majority of the PNSSP isolates, which resulted in an estimated PCV10 coverage of 94 % among those infected with PNSSP.

One of the main limitations of this study is the fact that all *S. pneumonia*e were isolated from meningitis cases. Information regarding other IPD manifestations in our region is often limited or unavailable. Although all of the pneumococcal isolates included in this study originated from only one hospital, Hospital Couto Maia is the state reference hospital in the city of Salvador, with about 95 % of the meningitis reports from the region originating from that site [12].

## Conclusions

Our results show sustained reductions in pneumococcal meningitis cases in the Metropolitan region of Salvador from 1996 to 2012, with a decrease of 82 % in the incidence of cases due to penicillin nonsusceptible pneumococci. In this setting, 94 % of PNSSP isolates were serotypes represented in the 10-valent pneumococcal conjugate vaccine. Although nationwide PCV10 vaccination just started in 2010, PCV7 has been available in private clinics and CRIEs nationwide since 2001, which may have contributed to the observed reductions on the incidence of pneumococcal meningitis cases. Furthermore no new serotype or genotype was observed after PCV10 implementation. However, the circulation of non-vaccine PNSSP types as well as capsule switching may compromise the effect of the conjugate vaccine in the future and highlights the need for the constant surveillance of circulating PNSSP isolates.

## Abbreviations

PCV10: Pneumococcal conjugate vaccine ten valent
PCV7: Pneumococcal conjugate vaccine seven valent
PCV13: Pneumococcal conjugate vaccine thirteenvalent
PNSSP: Penicillin non-susceptible*Streptococcus pneumoniae*
MIC: Minimal Inhibitory Concentration
PFGE: Pulsed-Field Gel Electrophoresis
MLST: Multi Locus Sequence Typing
CSF: Cerebrospinal Fluid
CRIE: Special Immunobiological Reference Center
